# Hepatic lipopolysaccharide binding protein partially uncouples inflammation from fibrosis in MAFLD

**DOI:** 10.1172/JCI179752

**Published:** 2024-09-03

**Authors:** Dan Wang, Ania Baghoomian, Zhengyi Zhang, Ya Cui, Emily C. Whang, Xiang Li, Josue Fraga, Rachel Ariana Spellman, Tien S. Dong, Wei Li, Arpana Gupta, Jihane N. Benhammou, Tamer Sallam

**Affiliations:** 1 David Geffen School of Medicine at UCLA, Los Angeles, California, USA.; 2 Division of Computational Biomedicine, Biological Chemistry, UCI, Irvine, California, USA.

**Keywords:** Genetics, Hepatology, Cholesterol, Fibrosis, Hepatitis

**To the Editor:** Metabolic dysfunction–associated fatty liver disease (MAFLD), is a heterogeneous spectrum liver disorder affecting 20% of the population. MASH is an advanced form of MAFLD associated with inflammation and fibrosis and can progress to cirrhosis. Scarring of liver tissue is a strong predictor of poor clinical outcomes, and multiple factors act synergistically to license fibrosis. Of particular interest is the role of inflammation, since the “multiple hit” model of MASH implies that inflammation incites fibrosis (1). Thus, targeting inflammation has been proposed to combat hepatic fibrosis. Surprisingly, treatment with the C-C chemokine receptors (CCR) antagonist cenicriviroc failed to meaningfully change fibrosis in a recent Phase III clinical trial (2). These results and others have raised questions about the therapeutic potential of targeting inflammation in MASH and our understanding of sequential events leading to the progression of MASH.

To investigate the interplay between innate immune responses and fibrosis, we explored a role for LBP in MAFLD. LBP is known to be induced in response to inflammatory signaling and facilitates immune cell recruitment and function (3, 4). Feeding mice diets known to induce MAFLD or treatment with LPS increased circulating LBP (Figure 1A and Supplemental Figure 1, A–D; supplemental material available online with this article; https://doi.org/10.1172/JCI179752DS1). *Lbp* is expressed in different tissues, but the main source of circulating LBP levels remains unknown. *Lbp* expression is highest in the liver and specifically parenchymal cells (Supplemental Figure 1, E and F), which led us to the hypothesize that hepatocytes are key contributors to circulating LBP. To explore this, we generated *Lbp*^fl/fl^ mice (Supplemental Figure 1G) and observed that hepatocyte-specific loss of LBP using an AAV-TBG-Cre approach completely abolished circulating LBP levels (Figure 1B). In addition, immune reconstitution of WT or *Lbp*^–/–^ bone marrow (4) on a hyperlipidemic background did not show changes in circulating LBP levels (Figure 1C). Collectively, these results suggest that circulating LBP is predominantly dictated by hepatocytes.

To understand the role of hepatocyte LBP in MAFLD, we fed hepatocyte-specific LBP knockout mice (L-KO) or controls (WT) FPC (rich in fructose, palmitate, and cholesterol) diet. We did not observe differences in hepatocyte lipid droplet, lipid species composition (Figure 1, D–F), animal weight, percent fat, and liver weight between groups (Supplemental Figure 2, A–D). LBP deficiency led to a significant reduction in inflammatory cells (Figure 1, D and E) compared with controls, although we did not observe differences in fibrosis (Figure 1, D and E and Supplemental Figure 2E). In line with the above results, gene expression analysis showed a reduction in inflammatory markers (Figure 1G) in L-KO compared with controls without changes in fibrosis (Figure 1H and Supplemental Figure 2F) or lipid metabolism genes (Supplemental Figure 2, G and H). Further, infiltrating monocytes/macrophages were reduced in L-KO mice compared with controls as shown by F4/80^+^CLEC4F^–^ (Figure 1I) and Ly6C^+^ staining (Supplemental Figure 2E), along with a significant reduction in circulating and liver inflammatory markers (Supplemental Figure 2, I and J). Transaminases were significantly elevated in controls compared with L-KO mice (Figure 1J), suggesting that the observed change in inflammation meaningfully impacts liver steatohepatitis. Taken together, the results suggest that loss of LBP reduces inflammatory activation in MAFLD independent of hepatic lipid composition and without altering fibrosis.

To confirm the influence of LBP deficiency on hepatic immune cell composition, we performed single-nucleus RNA-Seq (snRNA-Seq) on livers from WT and L-KO mice. Integrated transcriptomic analysis revealed distinct populations of liver cell-type clusters (Figure 1, K and L and Supplemental Figure 2K). No differences were seen in the expression of key fibrosis genes in stellate cells (Supplemental Figure 2L). The Immune 1 cluster included kupffer cells, neutrophils, and dendritic cells, as suggested by high cell type-specific markers including *Adrge1* (Supplemental Figure 2K). Major changes between WT and L-KO centered in the Immune 2 cluster (Figure 1, K and L). This cluster was enriched in recruited macrophage populations expressing low/intermediate macrophage markers including *Adrge1* (F4/80) and *Csf1r* (Supplemental Figure 2K). Further analysis of this cluster revealed remarkably distinct populations (Figure 1M, upper left). The L-KO subcluster showed enrichments of noninflammatory macrophage markers including *Vsig4*, *Cd5l*, and *Clec4f* (Figure 1M) and antiinflammation genes like *Lrg1* and *Gna15* (Supplemental Figure 2M), whereas WT mice exhibited higher levels of *Gpnmb*, which defines proinflammatory macrophages (Figure 1M).

To confirm the impact of LBP on hepatic scarring, we treated WT or L-KO mice with carbon tetrachloride (CCl_4_) and did not observe differences in fibrosis (Supplemental Figure 3, A–E). To translate our findings to human MAFLD, we first explored the expression of human *LBP* from GTEx. Human *LBP* is dominantly expressed in the liver and specifically hepatocytes (Supplemental Figure 3, F and G). In a cohort of MASH patients, we found that hepatic *LBP* expression strongly segregates the degree of liver inflammation but not steatosis or fibrosis (Figure 1N). GWAS showed that coding mutations in *LBP* are strongly associated with circulating markers of inflammation known to be important in MAFLD-like serum IL-15 levels (*P* value 8 × 10^–163^) (Supplemental Figure 3H). We confirmed a positive correlation between circulating IL15RA and LBP levels in an independent cohort of MASH patients (Figure 1O). Taken together, our results suggest that the human LBP may be protective against hepatic inflammation with minimal impact on fibrosis.

In summary, we uncovered what is, to our knowledge, a previously unidentified role for hepatic LBP in MAFLD. We found that loss of LBP reduced inflammation along with macrophage recruitment markers, but these changes were not sufficient to reduce hepatic fibrosis. Since hepatic scarring is a crucial driver of liver disease–related morbidity and mortality, our findings have important implications for approaches that aim to target inflammation to reduce fibrosis.

## Supplementary Material

Supplemental data

Unedited blot and gel images

Supporting data values

## Figures and Tables

**Figure 1:**
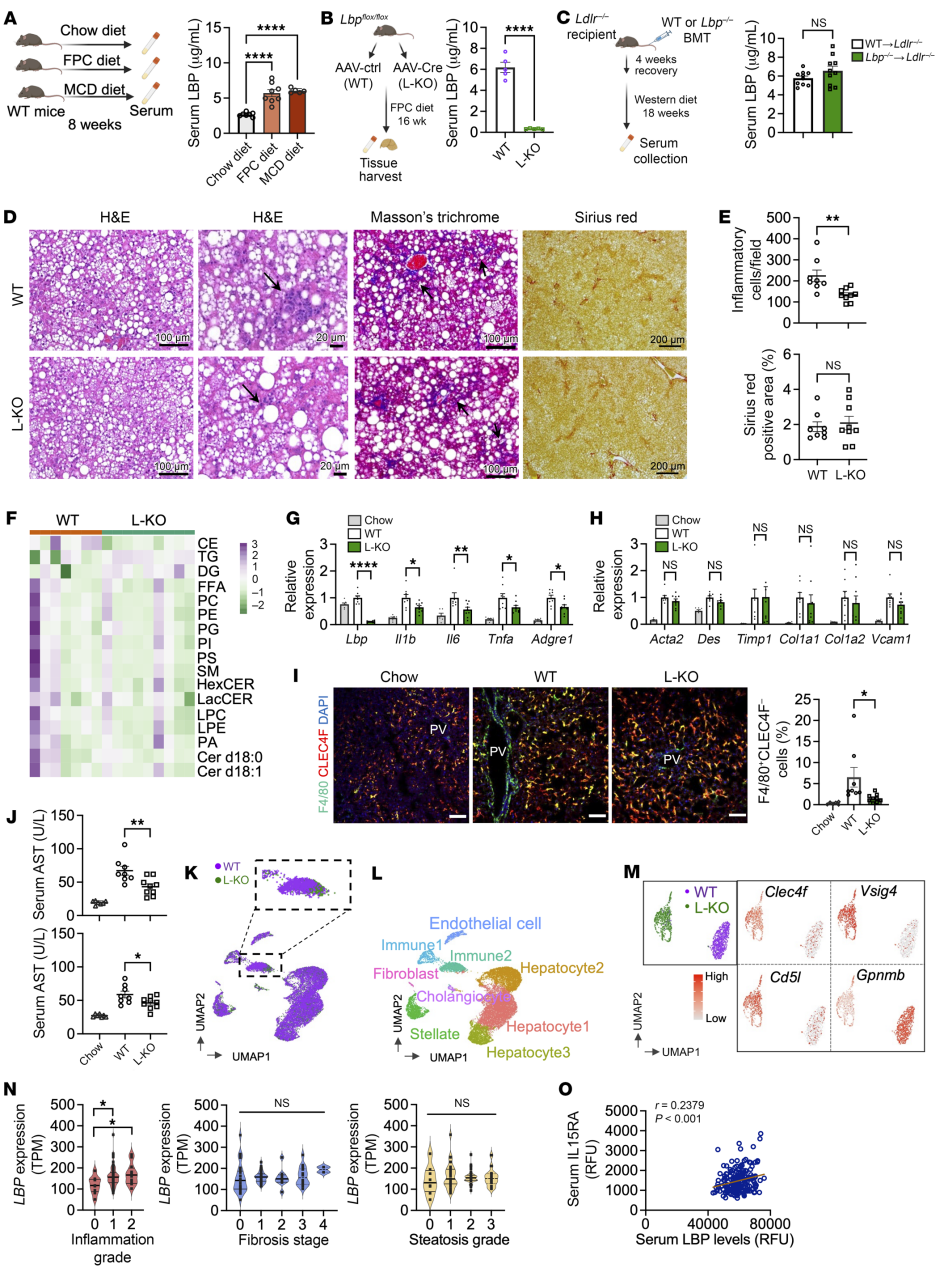
Hepatic LBP deficiency reduces inflammation but not fibrosis. (**A**–**C**) Serum LBP as indicated. (*n* = 5–8 in **A**, *n* = 5 in **B**, and *n* = 10 in **C**). (**D**) Liver H&E, Masson’s trichrome and Sirius red staining (arrows indicate inflammatory cells). (**E**) Quantification of inflammatory cells and fibrosis. (**F**) Liver lipidomics heatmap. (**G** and **H**) RT-qPCR from liver. (**I**) Liver IHC, scale bar: 100 μm. (**J**) Serum ALT/AST. (*n* = 8–9 in **E**–**J**). (**K** and **L**) UMAP plot and population annotation of snRNA-seq from liver. (**M**) Representative genes from the Immune 2 cluster. (**N**) RNA-Seq from human liver. (**O**) Correlation between circulating LBP and serum IL15RA in human MASH cohort. Data represent mean ± SEM. *P* value calculated by unpaired 2-tailed *t*-test (**B**, **C**, and **E**); 1-way ANOVA (**A**, **I**, **J**, and **N**); and 2-way ANOVA (**G** and **H**). *P < 0.05; **P < 0.01; ****P < 0.0001.

## Version 1 07/26/2024

In-Press Preview

## Version 2 09/03/2024

Electronic publication
