# Supplementary material for: Hepatic lipopolysaccharide binding protein partially uncouples inflammation from fibrosis in MAFLD
Source: J Clin Invest. 2024 Sep 3;134(17):e179752. doi: 10.1172/JCI179752 (PMC11364399; doi:10.1172/JCI179752)
Supplement: Supplemental data [file jci-134-179752-s049.pdf]

Supplemental Material

Methods

Sex as a biological variable

We did not observe differences between male and female mice in pilot studies and male mice were used in the presented data.

Animals and diets

All mice used in this study were housed in pathogen-free rooms with temperature monitors and a 12-hour light/12-hour dark cycle. Mice were generated from the EUCOMM/KOMP-CSD 'Knockout-First' C57BL/6 ES cell resource to get a whole-body knock out *Lbp* mice. Then they crossed with the FLPe knock-in mouse strain (JAX 009086) to generate the *Lbp*$^{flox/flox}$ mice. Hepatocyte-specific LBP knockout mice were injected with AAV8-TBG-Cre (L-KO) or AAV8-TBG-Control (WT) (Addgene 107787). *Apoe* (B6.129P2-*Apoe*^{tm1Unc}/J, 002052) and *Ldlr* (B6.129S7-*Ldlr*^{tm1Her}/J, 002207) mice are from the Jackson Laboratory. The diets used were the standard chow diet (Research Diets), FPC-MASH diet (Envigo, TD.190142), Western diet (Research Diets, D12079B), MCD diet (Research Diets, A02082002B), and mice were fed as indicated. The bone marrow transplantation was performed previously (1). Intraperitoneal injection of lipopolysaccharide (LPS) was done at 1.5mg/kg for a single dose or daily injection for up to 7 days. For CCl_4-induced liver fibrosis, L-KO and WT mice were IP injected biweekly for 4 weeks with 0.5 µl/g body weight of CCl_4 (Sigma), which was dissolved in corn oil at a ratio of 1:3.

Histopathological analysis and immunofluorescence

Hematoxylin and eosin (H&E) staining and Masson's trichrome staining of liver sections were prepared and stained from paraffin-embedded sections and performed by the Translational

Pathology Core Laboratory (TPCL) at UCLA. For Sirius red staining, the embedded samples were cut into 4 µm-thick sections and stained with Sirius Red (saturated picric acid containing 0.1% DirectRed 80, Sigma-Aldrich). For assessing the fibrosis in Masson's trichrome staining and Sirus red staining, the sections were evaluated using x5 magnification. 4~5 different microscopic fields were selected randomly in each individual liver section. The inflammatory cells in H&E staining were quantified as the number of mononuclear cells per field using a 20x objective. For each slide, 3~5 different microscopic fields were selected randomly in each individual liver section. The inflammatory cells per field and fibrosis contents from each mouse were quantified with ImageJ software. For immunofluorescence, liver sections were labeled with primary antibodies overnight at 4 °C, followed by incubation with a fluorophore-conjugated secondary antibody for 1 hour. Primary antibodies are anti-F4/80 at a 1:400 dilution, Abcam, ab6640, clone CI:A3-1; anti-alpha-smooth muscle actin monoclonal antibody at a 1:400 dilution, eBioscience, clone 1A4; anti-Ly-6C at a 1:200 dilution, BD Pharmingen, Clone: HK1.4; and anti-CLEC4F/CLECSF13 antibody at a 1:200 dilution, R&D, AF2784. The stained sections were stained with DAPI and then mounted (Life Technologies, P36935).

Serum LBP ELISA

Collected serum samples were stored at -80 °C and brought to room temperature prior to ELISA assay. LBP ELISA was performed according to the manufacturer's instructions (HycultBiotech, HK205). Briefly, 100 µl of the diluted sample and standards were transferred to the appropriate wells. The plate was incubated at room temperature for one hour before being washed with 200 µl of dilution buffer. 100 µl of the tracer and 100 µl of the streptavidin-peroxidase were added in sequence and incubated for 1 hour in each reaction. After washing, the TMB substrate was

incubated for 20 minutes before adding stop solutions. The plate reader was set at 450 nm following the manufacturer instructions and within 30 minutes of adding the stop solution.

Liver lipidomics

For lipid quantification analysis, approximately 80 mg of frozen liver was used. Samples were analyzed in UCLA Lipidomics Core on the Sciex Lipidyzer Platform using mass spectrometry methodology for targeted quantitative measurement of over 1400 lipid species across 17 lipid subclasses (2, 3). The measured lipid classes include cholesterol esters (CE), ceramides (Cer d18:1), diacylglycerol (DG), dihydroceramides (Cer d18:0), free fatty acids (FFA), hexosylceramides (HexCER), lactosylceramide (LacCER), lysophosphatidylcholine (LPC), lysophosphatidylethanolamine (LPE), phosphatidic acid (PA), phosphatidylcholine (PC), phosphatidylethanolamine (PE), phosphatidylglycerol (PG), phosphatidylinositol (PI), phosphatidylserine (PS), sphingomyelin (SM), and triglyceride (TG). Each quantitative value was normalized to milligrams of corresponding samples.

Gene expression analysis (RT-qPCR)

Liver samples were homogenized and prepared for RNA isolation using TRIzol (Invitrogen) (4). cDNA was synthesized using reverse transcription enzyme with oligo dT and random hexamers. Quantitative real-time PCR (qRT-PCR) was performed using SsoAdvanced Univ SYBR Green (Bio-Rad 1725275) to analyze gene expression. The results were normalized to housekeeping gene *Rplp0*. The primer sequences for qRT-PCR are listed in Supplemental Table 1.

Immunoblotting

About 100 mg of the protein from the frozen liver tissue samples was isolated and extracted. The samples were dounced using 300 µl of RIPA buffer (Boston Bioproducts) and centrifuged for

15 minutes at 10,000 g. The pure protein layer was extracted without disturbing the pellet or fat layers followed by protein quantification using the BCA assay. Equal amounts of protein were loaded into the 4-12% NuPAGE Bis-Tris Gel (Invitrogen) and the gel was run at 130 volts for 1 hour and 30 minutes. Protein was transferred from the gel to the PVDF membrane (0.45μm) at 100 volts and then blocked in a 5% milk solution. The primary antibodies were anti-Phospho-p44/42 MAPK (Erk1/2) (Thr202/Tyr204) (Cell Signaling Technology, 9101) and anti-GAPDH (GeneTex, GTX627408). Protein quantification was performed using ImageJ.

Serum cholesterol and liver function tests

The serum ALT and AST levels were measured according to the manufacturer's instructions (Teco Diagnostics, NC9851324 for AST and NC9851323 for ALT tests). Serum total cholesterol levels and triglyceride levels were tested using Wako kits as we previously described (5, 6).

Single nuclei isolation from liver samples of FPC diet fed mice

For the snRNA-seq, we isolated nuclei from male mice livers fed with FPC diet for 16 weeks using a protocol modified from previous publications (7, 8). Briefly, frozen livers pooled from 4 mice in each group were used for downstream studies. 4 ml of lysis buffer (10 mM Tris-HCl, 10 mM NaCl, 1 mM $MgCl_2$, 0.1% IGEPAL and 0.3 U/μL RNase inhibitor) were added to 80 mg of the dounced liver samples followed by gentle homogenization. After filtering through 30 μm cell strainer, 4 ml of WRB buffer (PBS, 2% BSA, and 0.2 U/μL RNase inhibitor) was added and centrifuged at 500 g for 5 min at 4 °C. The lipid layer was removed, and pellet resuspended in WRB buffer and filtered again with 40 μm Flowmi cell strainer. Nuclei were pelleted by centrifuging at 500 g for 5 min at 4 °C again and resuspended in WRB buffer. To assess the

quality of the isolated nuclei, we stained the nuclei with Hoechst stain for evaluation and counting.

10x Genomics single-nucleus RNA sequencing

We performed 10x Genomics single-nucleus RNA sequencing at UCLA Technology Center for Genomics & Bioinformatics Core immediately after the nuclei isolation. Single cell RNA sequencing was prepared using 10X Genomics' Chromium Next GEM Single Cell 3' Kit v3.1 following the manufacturer's protocol. The 10X 3'GEX libraries were sequenced on the Illumina NovaSeq6000 platform at a read length of 28x 90 and sequencing depth of 300-400 million reads per sample.

Single-nucleus RNA-sequencing analysis

Sequencing data de-multiplexing was performed using CellRanger mkfastq software v7.2.0 (10X Genomics). Sequenced reads in FASTQ format were aligned and analyzed via the CellRanger count pipeline v7.2.0 (10X Genomics) using the refdata-gex-mm10-2020-A mouse (10X Genomics) reference database under default settings. The R package Seurat was used to cluster the cells in the merged matrix (9). Subclusters were annotated using the marker genes according to previous publications (10-12).

Statistical analysis

Non-paired Student's *t*-test or ANOVA were used to determine the statistical significance defined at $P < 0.05$. Data was shown as mean, and the error bar indicates SEM. Group sizes were based on statistical analysis of variance and prior experience with similar *in vivo* studies. *P<0.05; ** P<0.01; *** P<0.001; **** P<0.0001.

Study approval

All experiments in this study were approved by the UCLA Institutional Animal Care and Research Advisory Committee, and performed in accordance with the recommendations in the Guide for the Care and Use of Laboratory Animals of the National Institutes of Health.

Data availability

The sequencing data generated in this study can be accessed under GSE254626. Date from publicly available datasets were from the Gene Expression Omnibus at the National Center for Biotechnology Information (NCBI) or the Genotype-Tissue Expression (GTEx) project. The MASH patient dataset with disease grading can be found under GEO accession number GSE130970 and genome-wide association study is available from previous publications (13). Serum protein analysis is from the study of proteo-transcriptomic map of non-alcoholic fatty liver disease (14). Mouse liver single-cell RNA-seq data (Tabula Muris) (15) are available from the Gene Expression Omnibus (GSE109774). Human liver cellular landscape by single-cell RNA-seq is from GSE115469 (12). Values for all data points in graphs are reported in the Supporting Data Values file.

Acknowledgments

We thank all members of the Sallam laboratory and UCLA Lipidomics Core. The confocal microscopy was performed at the California NanoSystems Institute of Advanced Light Microscopy/Spectroscopy Facility at UCLA. We thank Dr. Marcus Alvarez and Dr. Paivi Pajukanta for providing a protocol for the single-nuclei isolation. T.S. is supported by National Institute of Health (NIH) grants (DK118086, HL139549, HL149766), an American Heart Association Transformational Project grant and Burroughs Wellcome Fund Career Award for Medical Scientists. D.W. is supported by American Heart Association postdoctoral fellowship (906049).

A.G. is supported by R01 MD015904, K23 DK106528, R03 DK121025, ULTR001881/DK041301 (UCLA CURE/CTSI Pilot and Feasibility Study). Scientific schematics were created with BioRender.com.

References

1. Sallam T, Ito A, Rong X, Kim J, van Stijn C, Chamberlain BT, et al. The macrophage LBP gene is an LXR target that promotes macrophage survival and atherosclerosis. *J Lipid Res.* 2014;55(6):1120-30.
2. Salisbury DA, Casero D, Zhang Z, Wang D, Kim J, Wu X, et al. Transcriptional regulation of N(6)-methyladenosine orchestrates sex-dimorphic metabolic traits. *Nat Metab.* 2021;3(7):940-53.
3. Zhang Z, Feng AC, Salisbury D, Liu X, Wu X, Kim J, et al. Collaborative interactions of heterogenous ribonucleoproteins contribute to transcriptional regulation of sterol metabolism in mice. *Nat Commun.* 2020;11(1):984.
4. Zhang Z, Cui Y, Su V, Wang D, Tol MJ, Cheng L, et al. A PPARgamma/long noncoding RNA axis regulates adipose thermoneutral remodeling in mice. *J Clin Invest.* 2023;133(21).
5. Sallam T, Jones MC, Gilliland T, Zhang L, Wu X, Eskin A, et al. Feedback modulation of cholesterol metabolism by the lipid-responsive non-coding RNA LeXis. *Nature.* 2016;534(7605):124-8.
6. Sallam T, Jones M, Thomas BJ, Wu X, Gilliland T, Qian K, et al. Transcriptional regulation of macrophage cholesterol efflux and atherogenesis by a long noncoding RNA. *Nat Med.* 2018;24(3):304-12.
7. Alvarez M, Benhammou JN, Darci-Maher N, French SW, Han SB, Sinsheimer JS, et al. Human liver single nucleus and single cell RNA sequencing identify a hepatocellular carcinoma-associated cell-type affecting survival. *Genome Med.* 2022;14(1):50.

8. Machado L, Geara P, Camps J, Dos Santos M, Teixeira-Clerc F, Van Herck J, et al. Tissue damage induces a conserved stress response that initiates quiescent muscle stem cell activation. *Cell Stem Cell.* 2021;28(6):1125-35 e7.

9. Hao Y, Hao S, Andersen-Nissen E, Mauck WM, 3rd, Zheng S, Butler A, et al. Integrated analysis of multimodal single-cell data. *Cell.* 2021;184(13):3573-87 e29.

10. Xiong X, Kuang H, Ansari S, Liu T, Gong J, Wang S, et al. Landscape of Intercellular Crosstalk in Healthy and NASH Liver Revealed by Single-Cell Secretome Gene Analysis. *Mol Cell.* 2019;75(3):644-60 e5.

11. Kolodziejczyk AA, Federici S, Zmora N, Mohapatra G, Dori-Bachash M, Hornstein S, et al. Acute liver failure is regulated by MYC- and microbiome-dependent programs. *Nat Med.* 2020;26(12):1899-911.

12. MacParland SA, Liu JC, Ma XZ, Innes BT, Bartczak AM, Gage BK, et al. Single cell RNA sequencing of human liver reveals distinct intrahepatic macrophage populations. *Nat Commun.* 2018;9(1):4383.

13. Gudjonsson A, Gudmundsdottir V, Axelsson GT, Gudmundsson EF, Jonsson BG, Launer LJ, et al. A genome-wide association study of serum proteins reveals shared loci with common diseases. *Nat Commun.* 2022;13(1):480.

14. Govaere O, Hasoon M, Alexander L, Cockell S, Tiniakos D, Ekstedt M, et al. A proteo-transcriptomic map of non-alcoholic fatty liver disease signatures. *Nat Metab.* 2023;5(4):572-8.

15. Tabula Muris C, Overall c, Logistical c, Organ c, processing, Library p, et al. Single-cell transcriptomics of 20 mouse organs creates a Tabula Muris. *Nature.* 2018;562(7727):367-72.

Supplemental Figure 1

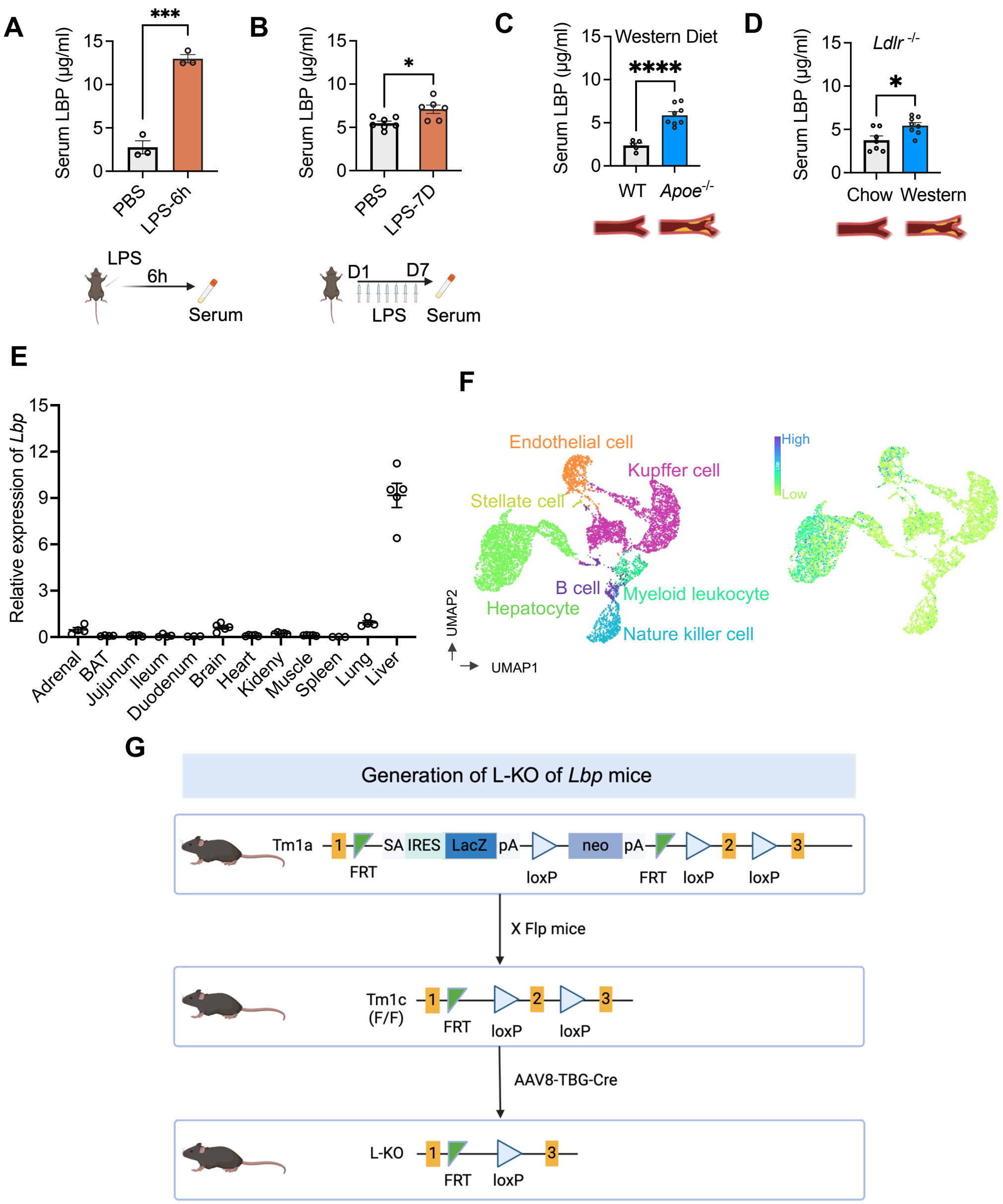

Supplemental Figure 1. Circulating LBP is regulated with lipid-rich diet. (**A**) Serum LBP 6 hours after LPS treatment (*n*= 3 per group). (**B**) Serum LBP following 1 week of LPS (*n*= 6~7 per group). (**C**) Serum LBP from *Apoe* KO mice compared with WT on Western diet for 4-6 weeks (WT: *n*= 5, *Apoe*$^{-/-}$: *n*= 8). (**D**) Serum LBP from *Ldlr* KO mice fed with the Western diet for 16 weeks compared with chow diet (*n*= 7~8 per group). (**E**) Gene expression of *Lbp* in a mouse tissue panel (*n*= 3~5 per group). (**F**) Mouse liver single-cell RNA-seq data. UMAP was produced by cellxgene using GSE109774 dataset. (**G**) Strategy for generating *Lbp*$^{flox/flox}$ mice. Data represent mean ± SEM. Non-paired Student's *t*-test was applied. *: $p < 0.05$; ***: $p < 0.001$.

Supplemental Figure 2

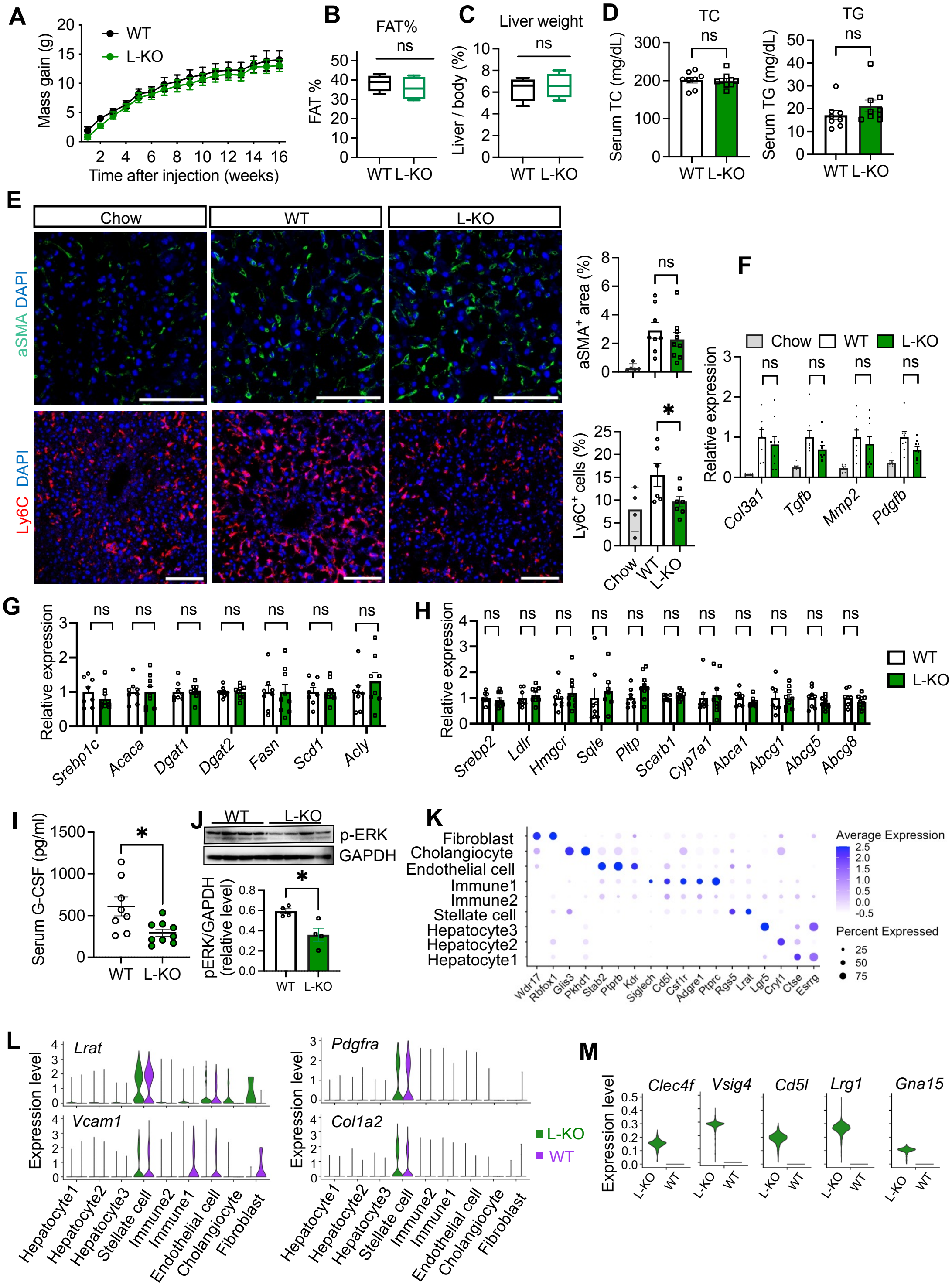

Supplemental Figure 2. Mice lacking hepatocyte LBP exhibit reduced liver inflammation but not fibrosis. (**A**) Body weight comparing WT and L-KO mice on FPC diet (*n*= 8~9 per group). (**B**) Fat mass between WT and L-KO (*n*= 5 per group). (**C**) Liver/body weight ratio from **B** (*n*= 5 per group). (**D**) Total cholesterol (TC) and total triglyceride (TG) (*n*= 8~9 per group). (**E**) aSMA staining and Ly6C staining from liver section related to experiments in Figure 1 (*n*= 8~9 per group). Bar, 100 µm. (**F**) Gene expression of fibrosis markers (*n*= 8~9 per group). (**G-H**) Gene expression measurement from mouse livers in WT and L-KO groups (*n*= 8~9 per group). (**I**) Serum G-CSF (*n*= 8~9 per group). (**J**) Western blot of Phosphorated ERK (p-ERK) from whole liver lysates (*n*=4 per group). Bar plot shows the quantification results using ImageJ. (**K**) Expression of marker genes from different clusters in snRNA-seq analysis in Figure 1. (**L**) Normalized expression of *Lrat, Vcam1, Pdgfra* and *Col1a2* in stellate cells under WT and L-KO group in snRNA-seq data. (**M**) Violin plots showing the expression of representative genes in WT and L-KO mice. Data represent mean ± SEM. Non-paired Student's *t*-test was applied except one-way ANOVA in supplemental Figure 2 (E). *: $p < 0.05$; ns: not significant.

Supplemental Figure 3

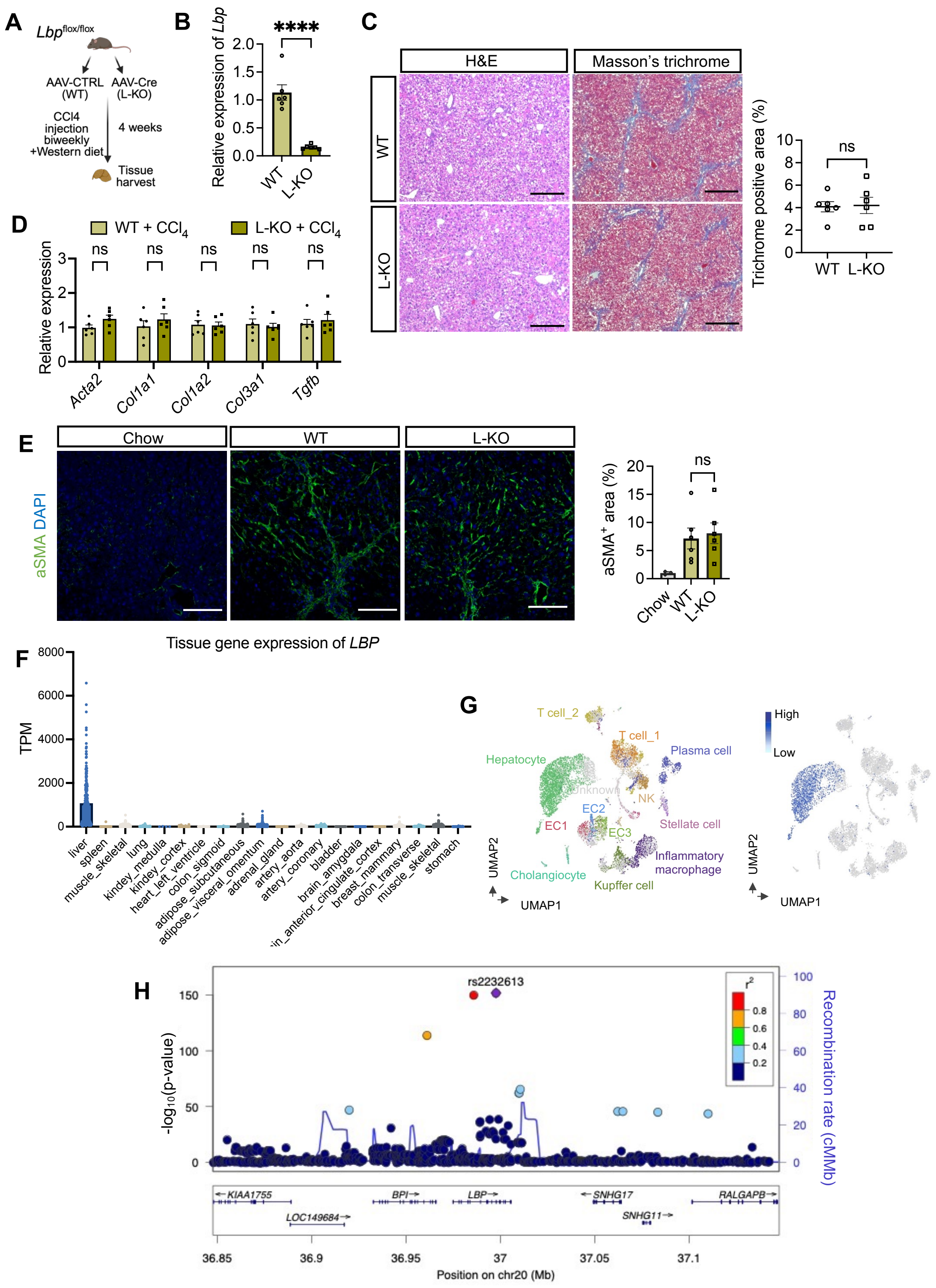

Supplemental Figure 3. Variants at human *LBP* are associated with inflammation. (**A**) Experimental design of CCl_4 treatment. (**B**) RT-qPCR results from liver in **A**. (*n*= 6 per group). (**C**) H&E staining and Masson's trichrome staining from liver sections. Quantification of Masson's trichrome staining area shown (*n*= 6 per group). Bar, 100 µm. (**D**) Gene expression of fibrosis markers in liver (*n*= 6 per group). (**E**) Staining of aSMA in liver section with quantification (*n*= 4 in chow, and *n*= 6 per group in treatment). Bar, 100 µm. (**F**) *LBP* expression in human tissues from GETx database. (**G**) Human liver single-cell RNA-seq. UMAP produced by Single Cell Expression Atlas using GSE115469 dataset. (**H**) LocusZoom plot showing genome-wide significant association observed in serum IL15 levels at *LBP*. Data represent mean ± SEM. Non-paired Student's *t*-test was applied for all except one-way ANOVA in Supplemental Figure 3 (E). ****: $p < 0.0001$; ns: not significant.

Supplemental Table 1: RT-qPCR primers

Primer name	Sequence
Rplp0-F	GGCCCTGCACTCTCGCTTTC
Rplp0-R	TGCCAGGACGCGCTTGT
Lbp-F	CCACAGATGGAGATCGAAGG
Lbp-R	ATCCCGGTAACCTTGCTGTT
Il6-F	CTTCACAAGTCGGAGGCTTAAT
Il6-R	GCAAGTGCATCATCGTTGTTC
Il1b-F	CTCCACCTCAATGGACAGAATATC
Il1b-R	GGGTGTGCCGTCTTTCATTA
Tnfa-F	CTTCTGTCTACTGAACTTCGGG
Tnfa-R	CAGGCTTGTCACTCGAATTTTG
Adgre1-F	ACCACAATACCTACATGCACC
Adgre1-R	AAGCAGGCGAGGAAAAGATAG
Acta2-F	ATGCTCCCAGGGCTGTTTTCCCAT
Acta2-R	GTGGTGCCAGATCTTTTCCATGTCG
Timp1-F	CTCAAAGACCTATAGTGCTGGC
Timp1-R	CAAAGTGACGGCTCTGGTAG
Des-F	CTAAAGGATGAGATGGCCCG
Des-R	GAAGGTCTGGATAGGAAGGTTG
Col1a1-F	GCTCCTCTTAGGGGCCACT
Col1a1-R	CCACGTCTCACCATTGGGG
Col1a2-F	GTAACTTCGTGCCTAGCAACA
Col1a2-R	CCTTTGTCAGAATACTGAGCAGC
Vim-F	TTTCTCTGCCTCTGCCAAC
Vim-R	TCTCATTGATCACCTGTCCATC
Scd1-F	GAACTTACAAGGCACGGCTG
Scd1-R	TCTGAGAACTTGTGGTGGGC
Fasn-F	CGGCTGCTGTTGGAAGTC
Fasn-R	CCTCTGAACCACTCACACCC
Acaca-F	AAAAGCGACATGAACACCGT
Acaca-R	AGTAAGTGTAGGGTCCCGGC
Acly-F	AGGTGGCCCCAACTATCAAG
Acly-R	AATGGCCGTCATGTGAGTTT
Dgat1-F	CCATACCCGGGACAAAGAC
Dgat1-R	AGAATCTTGCAGACGATGGC
Dgat2-F	TCTGTCACCTGGCTCAACAG

Primer name	Sequence
Dgat2-R	ATGAGGATGACACTGCAGGC
Abcg1-F	TCA CCC AGT TCT GCA TCC TCT
Abcg1-R	GCA GAT GTG TCA GGA CCG AGT
Ldlr-F	AGGCTGTGGGCTCCATAGG
Ldlr-R	TGC GGT CCA GGG TCA TCT
Srebp1c-F	GGAGCCATGGATTGCACATT
Srebp1c-R	GGCCCGGGAAGTCACTGT
Srebp1a-F	GGCCGAGATGTGCGAACT
Srebp1a-R	GGCCGAGATGTGCGAACT
Sqle-F	GCCTCTCAGAATGGTCGTCT
Sqle-R	CGCATGTCCCAGAATAAGGA
Hmgcr-F	CTTGTGGAATGCCTTGTGATTG
Hmgcr-R	AGCCGAAGCAGCACATGAT
Pdgfb-F	TAT GAA ATG CTG AGC GAC CA
Pdgfb-R	GGG TCA TGT TCA AGT CCA GC
Mmp2-F	CTA CGA TGA TGA CCG GAA GTG
Mmp2-R	CCA TCA GAG CTC CAG GGT C
Col3a1-F	CTGTAACATGGAAACTGGGGAAA
Col3a1-R	CCATAGCTGAACTGAAAACCACC
Tgfb-F	CTG CTG ACC CCC ACT GAT AC
Tgfb-R	AGT GAG CGC TGA ATC GAA AG
