# Supplementary material for: Hepatic lipopolysaccharide binding protein partially uncouples inflammation from fibrosis in MAFLD
Source: J Clin Invest. 2024 Sep 3;134(17):e179752. doi: 10.1172/JCI179752 (PMC11364399; doi:10.1172/JCI179752)

Full unedited blots:

Full unedited blot (left) and merged with markers (right) for Supplemental Figure 2J, GAPDH.

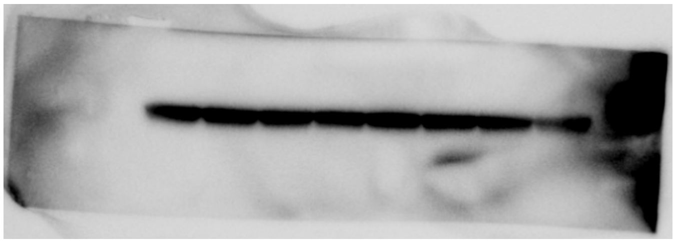

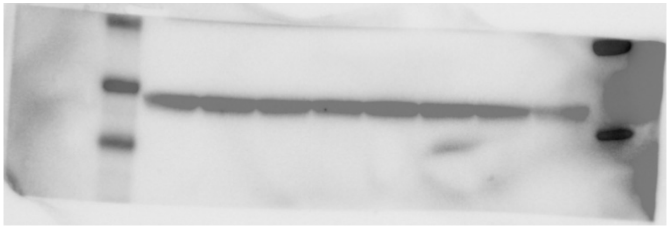

Full unedited blot (left) and merged with markers (right) for Supplemental Figure 2J, pERK.

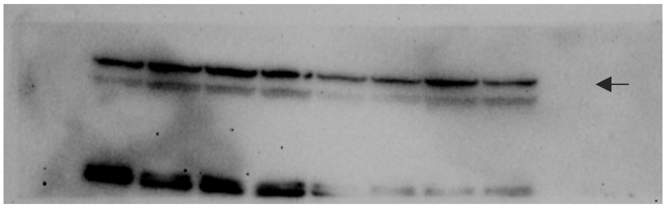

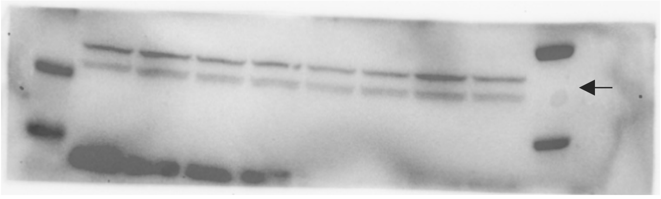

Supplement: Unedited blot and gel images [file jci-134-179752-s050.pdf]
